# Induction of colitis in mice with food allergen-specific immune response

**DOI:** 10.1038/srep32765

**Published:** 2016-09-08

**Authors:** Lin-Jing Li, Lu Zeng, Xiao-Xi Li, Li-Hua Mo, Xiao-Rui Geng, Peng-Yuan Zheng, Zhi-Gang Liu, Bai-Sui Feng, Ping-Chang Yang

**Affiliations:** 1 The Department of Gastroenterology, the Second Hospital, Zhengzhou University, Zhengzhou, China; 2 The Center of Allergy & Immunology, Shenzhen University School of Medicine, Shenzhen, China; 3 Longgang ENT Hospital, Shenzhen ENT Institute, Shenzhen, China; 4 The Department of Gastroenterology, the Fifth Hospital, Zhengzhou University, Zhengzhou, China

## Abstract

The pathogenesis of intestinal chronic inflammation is unclear. Food allergy plays an important role in the induction of intestinal inflammation. This study aims to test a hypothesis that food allergy initiates colitis. In this study, BALB/c mice were sensitized to a common food allergen, ovalbumin (OVA) with cholera toxin (CT) as an adjuvant. The colon epithelial barrier function was assessed with Ussing chamber technique. Expression of T cell immunoglobulin mucin domain molecule-4 (TIM4) in dendritic cells was evaluated by flow cytometry, RT-PCR and Western blotting. The results showed that allergen-related colitis was induced in mice as shown by heavy infiltration of inflammatory cells in the colon mucosa, loss of body weight of mice, increases in myeloperoxidase, tumor necrosis factor-α, interleukin-4, OVA-specific IgE in the colon tissue. The colon epithelial barrier function was markedly compromised in colitis group mice, which was mimicked by exposure the colon mucosa to CT in Ussing chamber. High frequency of TIM4^+^ dendritic cells was detected in the colon mucosa of colitis mice. Exposure of dendritic cells to CT markedly increased the expression of TIM4. We conclude that IBD-like inflammation can be induced in the mouse colon by the food allergen-related immune response.

The inflammatory bowel disease (IBD) includes Crohn’s disease and ulcerative colitis. The pathogenesis of IBD is unclear^1^. It is accepted that IBD is caused by aberrant immune response; probably induced by over reaction to the microbes or/and microbial products in the colon because most IBD occurs in the colon^2^. Genetic abnormality is associated with IBD^3^. Modification of epigenetics has been recognized in some IBD patients^4^, so does autoimmunity^4^. Association between ingesting offending food and IBD attack has been proposed^5^. However, the underlying mechanism has not been fully elucidated yet.

The immune phenotypes of IBD include T helper (Th)1 and Th2 types. The Th1 pattern inflammation of IBD is featured as over production of Th1 cytokines, such as interferon (IFN)-γ, tumor necrosis factor (TNF)-α, interleukin (IL)-1β and IL-17^6^. Some ulcerative colitis is featured as a Th2 pattern inflammation, in which high levels of Th2 cytokines play a major role in the inflammation of the colon mucosa^7^. It is generally accepted that food allergy is a Th2 pattern inflammation in the intestine^8^. Yet, although it has been proposed by clinical evidence that food allergy is associated with the pathogenesis of IBD^5^,^9^, the direct evidence has not been fully demonstrated.

Yet, the pathogenesis of food allergy is also unclear. Intestinal epithelial barrier dysfunction is proposed as one of the causative factors of food allergy^10^. Recent reports indicate that T cell immunoglobulin mucin domain molecule-4 (TIM4) plays a critical role in the initiation of Th2 polarization^11^,^12^. Whether the TIM4-related Th2 response is associated with IBD is unclear.

Based on the information above, we hypothesize that TIM4-related Th2 immune response is associated with the pathogenesis of IBD. In this study, with a well-established mouse model of food allergy, we induced colitis-like inflammation in mice. The data demonstrate that food allergy is one of the causative factors in the pathogenesis of IBD.

## Results

### Induction of colitis with food allergens in mice

**Figure 1:**
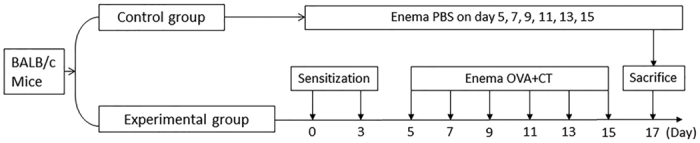
Procedures of inducing allergen-related colitis. BALB/c mice (6 mice per group) were subcutaneously injected with OVA (1 mg/mouse) mixed in 0.1 ml alum on day 0 and day 3 respectively. The mice were intrarectally introduced with OVA (1 mg/mouse) and CT (20 μg/mouse) as denoted in the figure.

**Figure 2:**
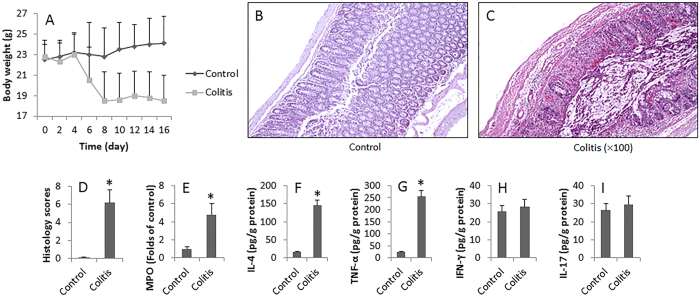
Induction of allergen-related colitis. BALB/c mice (12 mice/group) were treated with OVA/CT as described in Fig. 1. (**A**) The curves indicate the body weight of the mice. (**B**,**C**) The photomicrographs show the histology of the mouse colon (magnification = ×100). (**D**) The bars show the histology scores of (**B**,**C)**. (**E**–**J**) The bars indicate the levels of MPO (**E**) IL-4 (**F**) TNF-α (**G**) IFN-γ (**H**) and IL-17 (**I**) in the protein extracts of colon tissue. Data are presented as mean ± SD. *p < 0.01, compared with the control group. Samples from individual mice were processed separately.

Following the established procedures^13^, we repeatedly introduced OVA and CT into the mouse colon and induced immune inflammation in the colon (Fig. 1). The mice showed body weight loss (Fig. 2A), bloody feces, colon mucosal inflammation. The OVA-sensitized and challenged mice exhibited severe inflammation damage. The histological features of the colons of the control group mice were typical of a normal structure, whereas the inflamed colon of mice with OVA-induced colitis showed evidence of mucosal edema, crypt distortion, thickening of the colon wall, and high level of inflammatory cells infiltration (Fig. 2B–D). Increase in MPO, IL-4, TNF-α, but not IFN-γ or IL-17, in the colon was observed (Fig. 2E–I). The data demonstrate that the Th2-pattern inflammation is induced in the mouse colon.

### Allergen-specific immune response was detected in the colon

**Figure 3:**
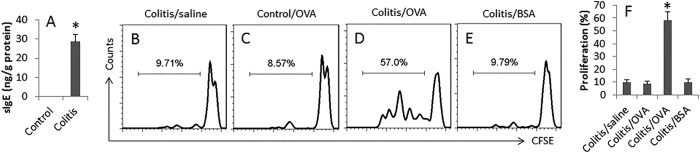
Assessment of OVA-specific immune response in the mouse colon. (**A**) the bars indicate the OVA-specific IgE levels in colon protein extracts (by ELISA). (**B**–**D**) the histograms indicate the frequency of proliferating CD4^+^ T cells in the mouse colon (by CFSE-dilution assay). (**E**) the bars indicate the summarized data of (**B**–**D)**. Samples were from 12 mice per group. Isolated CD4^+^ T cells of 3 mice were pooled as one sample. The data are representatives of 4 independent experiments. Data are presented as mean ± SD. *p < 0.01, compared with the control group. OVA: OVA (5 μg/ml) was added to the culture. BSA: BSA (5 μg/ml) was added to the culture (an irrelevant allergen used as a control).

We next assessed the specific immune response in the mouse colon. The protein extracts of the colon were analyzed by ELISA. The results showed that OVA-specific IgE was markedly increased in the colitis group, while it was below the detectable levels in the control group (Fig. 3A). We also isolated the lamina propria mononuclear cells (LPMC) from the mouse colon. CD4^+^ T cells were isolated from the LPMCs, labeled with CFSE and cultured with DCs for 3 days in the presence of specific allergen OVA or an irrelevant allergen BSA. The cells were analyzed by flow cytometry. The results showed that the CD4^+^ T cells from the colitis group markedly proliferated, but not in those stimulated with BSA (Fig. 3B–F), indicating that OVA-specific CD4^+^ T cells were induced in the colon.

### CT facilitates allergens to be transported across intestinal epithelial barrier

**Figure 4:**
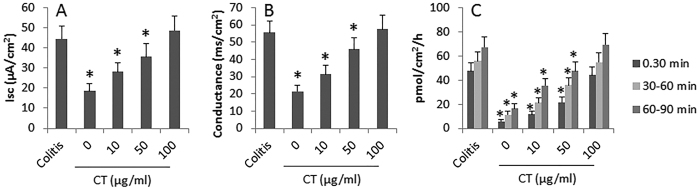
Exposure to CT compromises colon epithelial barrier function. The bars indicate the Isc (**A**) conductance (**B**) and colon epithelial layer permeability to OVA (**C**) Colitis: Samples were from colitis group. CT: Cholera toxin concentrations in the epithelial side of Ussing chambers, at where the samples were from control group. *p < 0.01, compared with the colitis group. Each group consists of 6 mice.

We next observed the effect of CT on facilitating OVA to be transported across colon epithelial barrier. Colon segments were collected from colitis mice and control mice and mounted on Ussing chambers. Isc, conductance and permeability to OVA (labeled with FITC) were recorded following our established procedures^10^. As shown by Fig. 4, the levels of Isc, conductance and permeability to OVA were significantly higher in the colitis group than in the control group (the “0” group). Exposure of colon tissue from control mice to CT in Ussing chambers markedly increased the levels of Isc, conductance and permeability to OVA in a CT dose-dependent manner. The results demonstrate that exposure to CT compromises the colon epithelial barrier function and facilitates the absorption of allergen.

### CT increases TIM4 expression by DCs in colon mucosa

**Figure 5:**
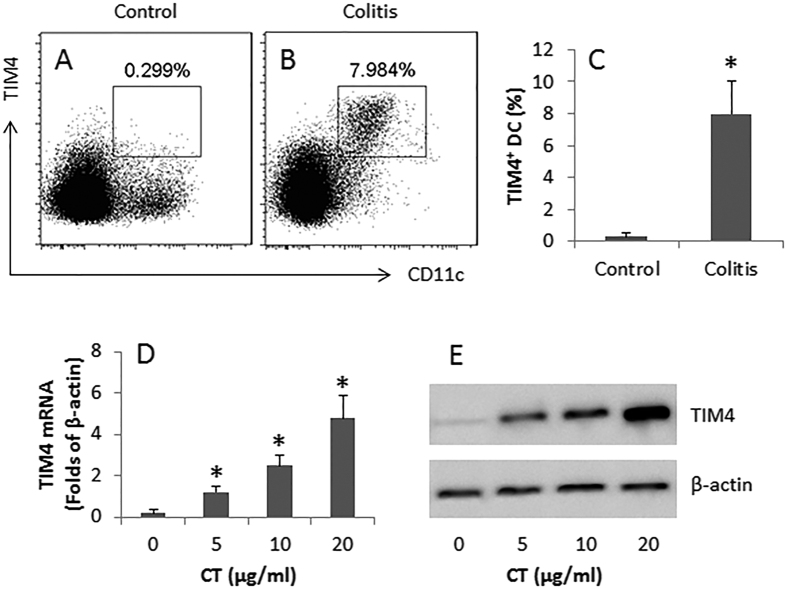
CT increases TIM4 expression in colon DCs. (**A**,**B**) the gated flow cytometry dot plots indicate the frequency of TIM-4^+^ DCs in the colon mucosa of mice (n = 6 per group) with or without colitis. (**C**) the bars indicate the summarized data of (**A**,**B)**. (**D**) the bars indicate the TIM4 mRNA levels in BMDCs after stimulated with CT in the culture for 48 h at graded doses (denoted on the X axis). (**E**) the immune blots indicate the protein levels of TIM4 in BMDCs after stimulating with CT in the culture. Samples from individual mice were processed separately. Data of bars are presented as mean ± SD. *p < 0.01, compared with the control group (**C**) or the dose “0” group (**D**). Data of (**D**,**E)** are representatives of 3 independent experiments. The full length gel graphs are presented in the relevant information.

TIM4 is a critical molecule in the initiation of Th2 polarization^12^. We then assessed the frequency of TIM4-expression of DCs in the colon mucosa of mice with or without colitis. As shown by flow cytometry data, the frequency of TIM4^+^ DCs was significantly higher in the colitis group than in the control group (Fig. 5A–C). Further analysis showed that DCs isolated from the intestine of colitis mice had high levels of TIM4 at both mRNA and protein levels (Fig. 5D,E).

## Discussion

It is recognized that IBD is a disease with multiple causative factors in the pathogenesis^1^. However, the detail mechanism of IBD is to be further understood. By using a well-established food allergy mouse model, we carried out this study to elucidate the role of food allergy in the pathogenesis of IBD. The results demonstrate that, in the presence of a specific adjuvant, repeatedly exposure to food allergens can induce IBD-like inflammation in the colon. The data show that, after treated mice with OVA and CT for 5 times, colitis was induced in mice, which was similar to human IBD, including body weight loss, heavy infiltration of inflammatory cells in the colon mucosa and high levels of proinflammatory cytokines in the colon mucosa extracts. Therefore, the present study has provided experimental evidence to have defined a link between food allergy and chronic intestinal inflammation such as IBD, a phenomenon noticed by physicians in the clinic, but shorting of experimental data to support, in many years ago^14^.

In this study, we observed that upon challenge with a specific antigen, OVA, the IBD-like inflammation was induced in the mouse colon; those mice were sensitized to OVA. As Joachim *et al*. reported, after ingestion of certain foods, the intestinal inflammation got worse in IBD patients^14^. Bartunkova found a fraction of IBD patients showed food allergen-specific IgE in the sera^15^, indicating at least a portion of IBD patients had IgE-mediated immune inflammation in the body, while whether these food allergen-specific IgE contribute to the chronic inflammation in the intestine in those patients remains to be elucidated. Although it is not convenient to verify this in IBD patients, the present data provide evidence that food allergy is sufficient to induce IBD-like inflammation in the mouse intestine.

Cytokines are the major inflammatory mediators. In this study, we assessed 4 representative CD4^+^ T cell cytokines in the colon mucosa in the experimental mice, including IL-4, TNF-α, IFN-γ and IL-17. We chose evaluating IL-4 because it is the signature Th2 cytokine. The results showed that the levels of IFN-γ and IL-17 were not increased in the colon mucosa, indicating that the inflammation is not the Th1 pattern. The data show that the levels of IL-4 were significantly increased in the colon mucosa, indicating a Th2 pattern inflammation was induced. We also observed high levels TNF-α in the colon mucosa, which further corroborates the results that food allergy is associated with the pathogenesis of IBD; TNF-α is a critical inflammatory factor in IBD because administration of anti-TNF-α can well alleviate the clinical symptoms of IBD^16^.

In this study, CT was used as an adjuvant to induce food allergy in the colon. Our previous studies indicate that CT facilitates the development of Th2 polarization^17^. Other investigators also use CT to develop food allergy animal models^18^. The present data show that CT markedly compromised the colon epithelial barrier function and increased the allergen OVA transportation across the colon epithelial barrier. This is in line with previous studies, in which we found that CT promotes the expression of claudin-2 expression in the intestinal epithelium to increase the paracellular permeability^19^.

The data also show another aspect of CT in the development of Th2 polarization in the colon mucosa. We detected higher frequency of TIM4^+^ DCs in the colon mucosa of colitis mice. Although we found this phenomenon in previous studies, in which we observed that CT increased TIM4 expression to facilitate the development of food allergy in mice^11^,^12^, while we observed that TIM4 was also associated with the initiation of an allergen-related inflammation in the colon mucosa. We also observed that p300 and STAT6 mediate the microbial product CT-induced TIM4 expression in DCs in a recent study^13^.

In summary, the present data show that an allergen-related IBD-like inflammation can be induced in the colon, in which DC-producing TIM4 plays a critical role.

## Materials and Methods

### Mice and ethic statement

Male BALB/c mice (6–8 week old) were purchased from the Guangdong Experimental Animal Center. The mice were maintained in a pathogen-free environment with accessing food and water freely. The experimental procedures were approved by the Animal Ethic Committee at Shenzhen University. All the experiments were performed in accordance with the approved guidelines.

### Development of allergen-related inflammation in the colon

#### Sensitization and challenge of mice

As shown by Fig. 1, mice were subcutaneously injected with ovalbumin (OVA; 1 mg/mouse) and 5 μg/ml cholera toxin (CT) in 0.1 ml saline on day 1 and day 3 respectively. From day 5 to day 15, each mouse were intrarectally introduced with 1 mg OVA and 20 μg in 0.1 ml saline (control mice were introduced with saline using as a control) every other day in the same procedures of the development of TNBS colitis mouse model which we reported recently^20^. Briefly, after fasting overnight, mice were anesthetized and a 4 cm long catheter (attached to a 1-ml syringe) was inserted 3.5 cm into the colon, where the colon mucosa was challenged with the specific antigen, OVA (1 mg/mouse in 0.1 ml saline; or saline alone for control mice). Body weight was recorded for each mouse every other day. The mice were sacrificed on day 17.

#### Colon histology

A segment of the colon was excised and fixed with 4% formalin overnight. The tissue was processed for paraffin sections. The sections were stained with hematoxylin and eosin. The tissue structure and inflammation of the colon were observed under a light microscope. To avoid the observer bias, the sections were coded; the observers were not aware of the code.

#### Microscopic inflammation scoring

The histological damage scoring of colon tissue sections was graded based on epithelial lesion (0, none damage; 1, some loss of goblet cells; 2, extensive loss of goblet cells; 3, some loss of crypts; 4, extensive loss of crypts) and infiltration (0, none infiltration; 1, infiltration around crypt bases; 2, infiltration spreading to muscularis mucosa; 3, extensive infiltration in the muscularis mucosa with abundant edema; 4, infiltration spreading to submucosa). The total histological grade ranged from a minimum of 0 to a maximum of 8.

#### Assessment of TNF-α, IFN-γ. IL-4, IL-17 and MPO in the colon tissue

A segment of the colon was homogenized in protein extraction buffer. The protein extracts were analyzed by ELISA with commercial reagent kits of IL-4, IL-17, TNF-α and IFN-γ (R&D Systems) following the manufacturer’s instructions. Myeloperoxidase (MPO) activity of the protein extracts was determined using a MPO assay kit (Sigma Aldrich) following the manufacturer’s instructions.

### Assessment of allergen-specific immune response in the colon

The allergen-specific IgE and allergen-specific CD4^+^ T cells in the colon were assessed using as the indicators of allergen-specific immune response in the colon. The colon was excised from control mice and colitis mice. A segment of the colon was processed for protein extraction. The protein was analyzed with a reagent kit (Juebo Biotech, Shanghai, China) to determine the OVA-specific IgE following the manufacturer’s instructions. On the other hand, lamina propria mononuclear cells (LPMC) were isolated from the colon tissue with our established procedures^17^. The cell viability of the isolated LPMC was assessed by Trypan blue exclusion assay. The viability of LPMC was greater than 96%. The CD4^+^ CD25^−^ T cells and CD11c^+^ dendritic cells (DC) were purified from LPMCs with magnetic cell sorting (MACS) reagent kits (Miltenyi Biotech) following the manufacturer’s instructions. The purity of the isolated cells was checked by flow cytometry. If the purity was below 95%, the cells were processed with the purification procedures again. The CD4^+^ T cells (labeled with CFSE) and DCs were cultured at a ratio of 10^5^ cells:2 × 10^4^ cells/well in the presence of OVA (5 μg/ml) and PMA (10 ng/ml)/ionomycin (0.5 μg/ml) for 3 days. The cells were analyzed by flow cytometry.

### Labeling OVA with FITC

OVA was labeled with FITC with a FITC-labeling kit (Shanghai Haoran Bioteck, Shanghai, China) following the manufacturer’s instructions. Briefly, for each 1 mL of OVA solution (1 mg/ml), 50 mL of FITC solution (1 mg/ml) was added very slowly with continuously stirring; and then incubated the reaction in the dark for 8 h at 4 °C. The reaction was stopped by adding NH_4_Cl to a final concentration of 50 mM, mixed for a while then incubated for 2 h at 4 °C. The unbound FITC was separated from the conjugate by gel filtration using a fine-sized gel matrix with an exclusion limit of 20 to 60 kDa.

### Assessment of colon epithelial barrier function

The short circuit current (Isc), conductance and OVA-flux of the colon epithelium were assessed using as indicators of the colon epithelial barrier function. Following our established procedures^10^, the colon segments were mounted on Ussing chambers. The Isc and conductance were recorded 30 min after the mounting. OVA-FITC was added to the epithelial side of Ussing chambers. Samples were collected from the serosal side at 30 min, 60 min and 90 min respectively. The fluorescence intensity of the samples was measured with a flourometer (PerkinElmer, UK). The results were calculated against the original amount added to the epithelial side and presented as pmol of OVA/cm^2^/h.

### Assessment of TIM4 expression in DCs

#### Assessment of TIM4^+^ DCs in colon mucosa by flow cytometry

LPMCs were prepared as described above. The LPMCs were stained with FITC-anti-CD11c antibody and PE-anti-TIM4 antibody or isotype IgE (eBioscience) following our established procedures^11^. The cells were analyzed by flow cytometry. The data were analyzed with software Flowjo. The readouts of isotype IgG stained cells were used as the gating reference.

#### Assessment of the effect of CT on TIM4 expression in DCs

Bone marrow DCs were prepared as we reported previously^21^. The DCs were stimulated with CT at graded concentrations (5, 10, 20 μg/ml) in the culture.

#### Assessment of TIM4 mRNA in DCs by real time quantitative RT-PCR (RT-qPCR)

DCs were collected from the culture. Total RNA was extracted with TRIzol reagents (Invitrogen). The cDNA was synthesized with the RNA and a reagent kit (Invitrogen) following the manufacturer’s instructions, and amplified by qPCR in a qPCR device (mini Opticon; Bio-Rad) with SYBR Green Master Mix (Invitrogen) and a pair of primer of TIM4 (gctgcttccaacaacagtca and gtgattggatgcaggcagag). β-actin was used as an internal control; its primers are gtgggaatgggtcagaagga and tcatcttttcacggttggcc. The results were calculated with the 2^−ΔCt^ method and presented as folds of β-actin.

#### Assessment of TIM protein in DCs by Western blotting

The total proteins were extracted from DCs, fractioned by SDS-PGAE and transferred onto a PVDF membrane. After blocking with 5% skim milk for 30 min, the membrane was incubated with a goat-anti-mouse TIM4 mAb (M-17; 1:500) overnight at 4 °C, followed by incubation with a rabbit-anti-goat IgG (1:10000; labeled with peroxidase) for 1 h at room temperature. Washing with TBST (Tris-buffered saline Tween 20) was performed after each time of incubation. The membrane was developed with ECL. The results were photographed with an image device (UVI Image Station, Cambridge, UK).

### Statistics

Data are presented as mean ± SD. Difference between 2 groups was determined by Student t test or ANOVA if more than two groups. P < 0.05 was set as a significant criterion.

## Additional Information

**How to cite this article**: Li, L.-J. *et al.* Induction of colitis in mice with food allergen-specific immune response. *Sci. Rep.* **6**, 32765; doi: 10.1038/srep32765 (2016).
